# Factors associated with hepatitis C in residents of Sao Paulo, Brazil

**DOI:** 10.1186/1742-4690-9-S1-P53

**Published:** 2012-05-25

**Authors:** Norma Farias, Umbeliana Barbosa de Oliveira, Débora Moraes Coelho, Iára de Souza, Claudia Afonso Binelli

**Affiliations:** 1 Epidemiology at Secretaria de Estado Da Saúde de São Paulo- Brazil, Sao Paulo, Brazil

## Introduction

Hepatitis C represents a global public health problem. The aim of the present study was to describe the epidemiological profile and to assess exposure variables associated with hepatitis C in residents of Sao Paulo, from the database of viral hepatitis at the National Databank of Major Causes of Morbidity.

## Material and methods

We analyzed 24,140 cases of hepatitis C notified in residents of the State of Sao Paulo, among the 46,969 bank records of viral hepatitis between 2007 and 2010. Suspected cases of hepatitis C have been confirmed by the presence of HCV RNA using reverse transcription-polymerase chain reaction (RT-PCR). The variables selected from the notifications files were sex, age, race, education, number of sexual partners, history of sexually transmitted diseases, HIV/AIDS, intravenous drugs use, inhaled or crack, injecting drugs, tattooing/piercing, acupuncture, blood/products transfusion, surgical, dental treatment, hemodialysis, transplantation and accidents with biological material. Factors associated with HCV infection were identified with univariate and multivariate Poisson regression and confidence intervals of 95%.

## Results

The detection rate of hepatitis C was about 15/100000 inhabitants. People aged 50 and over (PR=2,11;95% CI:1,96-2,27), history of blood transfusion (PR=1,41;95% CI: 1,34-1,48), intravenous drugs use (PR=1,33;95% CI: 1,25-1,42), inhalable drugs use or crack (PR=1,27( 1,20-1,35), HIV/AIDS (PR=1,20;95% CI:1,13-1,28), surgical treatment in the past (PR=1,16;95% CI:1,11-1,21) were the main factors independently associated with infection by hepatitis C virus.

## Conclusions

These findings reinforce the importance of preventing hepatitis C in vulnerable populations such as drug users and the implementation of drugs misuse related harm reduction programs targeted these segments.

